# Novel formulation for co-delivery of cinnamon- and cumin-loaded polymeric nanoparticles to enhance their oral bioavailability

**DOI:** 10.1007/s13205-023-03480-8

**Published:** 2023-01-27

**Authors:** Aditi Sangal, Sunita Rattan, Muni Raj Maurya, Kishor Kumar Sadasivuni

**Affiliations:** 1 Amity Institute of Applied Sciences, Amity University, Uttar Pradesh, Sector 125, Noida, 201313 India; 2 Center for Advanced Materials, Qatar University, Building H10, Zone 6, Office E133, PO Box 2713, Doha, Qatar

**Keywords:** Bioavailability, Drug delivery, Encapsulation, Polymeric nanoparticles, Sustained release

## Abstract

Nanobiotechnology has been an encouraging approach to improving the efficacy of hydrophobic bioactive compounds. The biologically active constituents present in herbal extracts are poorly absorbed, resulting in loss of bioavailability and efficacy. Hence, herbal medicine and nanotechnology are combined to overcome these limitations. The surface-to-volume ratio of nanoparticles is high and as the size is small, the functional properties are enhanced. The present study reports the synthesis of cinnamon and cumin (Ci–Cu) dual drug-loaded poly (D, L-lactide-co-glycolide) (PLGA) nanoparticles (NPs) to overcome the limitations of oral bioavailability and extend the effect of these drugs for alleviating health problems. The solvent evaporation method was adopted for the synthesis, and the as-prepared nanoparticles were characterized by Scanning electron microscopy (SEM), Fourier transform infrared (FTIR) spectroscopy, Transmission electron microscopy (TEM) and X-ray diffraction (XRD). The average size of the formed spherical Ci-Cu nanoparticles ranged between 90 and 120 nm. The encapsulation efficiency of the drug was found to be 79% ± 4.5%. XRD analysis demonstrated that cinnamon and cumin were amorphously scattered in the PLGA matrix. The FTIR bands showed no evident changes suggesting the no direct molecular interactions between the drug and the polymer. At pH 6.9, the release studies in vitro exhibited a burst initially followed by a tendency to obtain a slower steady release. The results indicated that the Cu-Ci dual drug-loaded polymeric NPs has drug release at a slower rate. The time taken for 25% release of drug in Ci-Cu-loaded PLGA NPs was twice as compared to cumin-loaded PLGA Nps, and three times compared to cinnamon-loaded PLGA NPs.

## Introduction

Spices are aromatic substances found in dried plant parts, mostly seeds, leaves, fruits, bark, roots, or other plants with a distinct smell. As all spices are obtained from plants, they have been generally recognized as safe (GRAS). Plant leaves, fruits, seeds and bark have polyphenols due to which they are added to increase flavor, preserve food or add color. However, spices can be used for functions more than just these in the foods to which they are added. Spices have usage as traditional medicine in various countries and play a great role because of their antioxidant, anti-inflammatory, antimicrobial, antimutagenic, antidiabetic and anti-cancerous activities (Yashin et al. 2017; Paswan 2021).

**Fig. 1:**
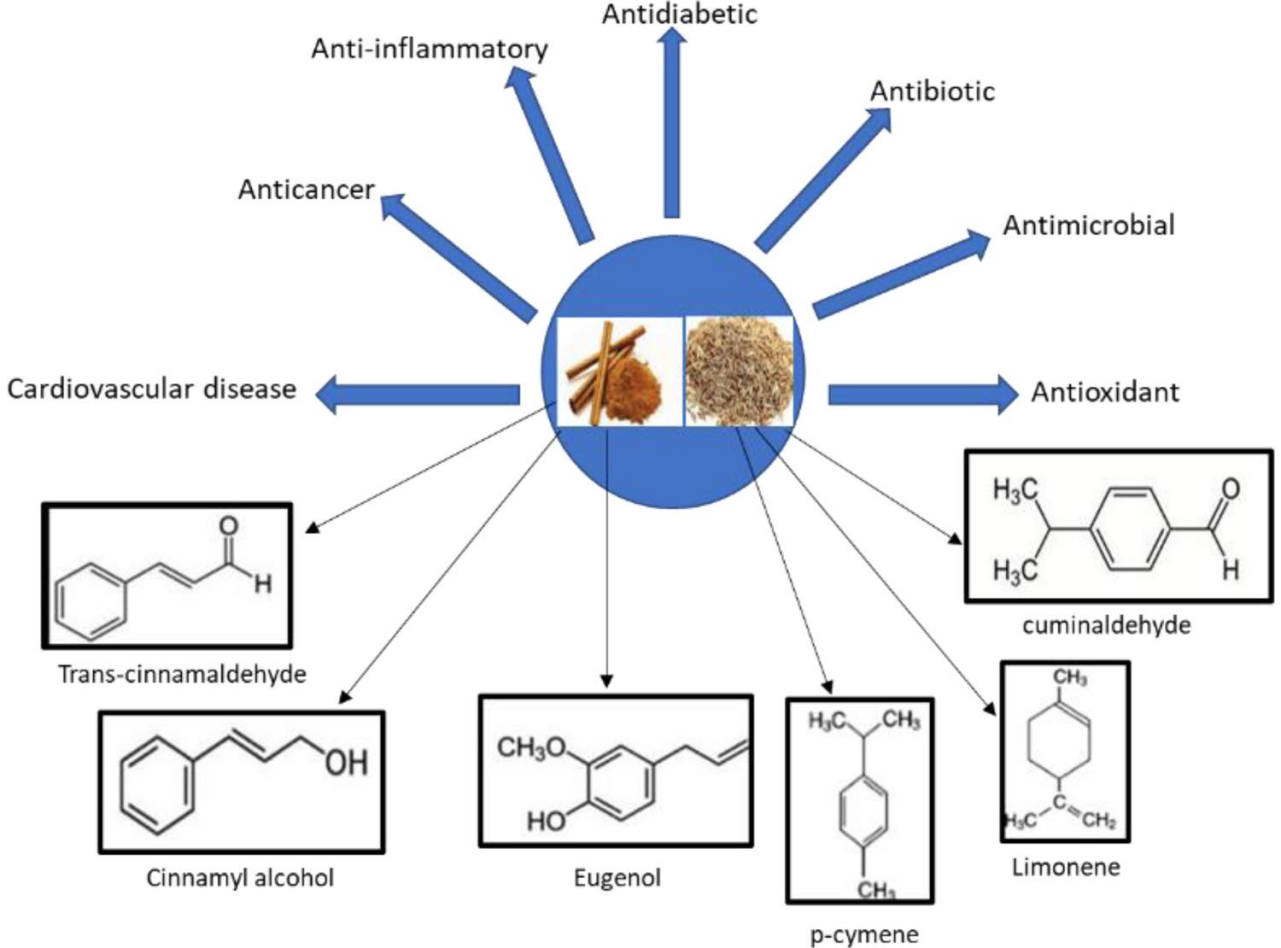
The active components in cinnamon and cumin focusing their therapeutic benefits

Cinnamon (Ci) is consumed frequently and is of different types, common or true cinnamon (C. *verum*, Cinnamomum *zeylanicum*,) and cassia (Cinnamomum *aromaticum*). 3-phenyl-2propenal (trans-cinnamaldehyde) is the key component in the bark oil of cinnamon, whereas, in oil obtained from leaf it is eugenol (Błaszczyk et al. 2021). Recent reported research suggests that cinnamon possess therapeutic effects including antioxidant (Błaszczyk et al. 2021; Gulcin et al. 2019; Anand et al. 2016; Kallel et al. 2019), antimicrobial (Doyle and Stephens 2019; Hamidpour et al. 2015; Abdallah et al. 2020; Ajay et al. 2020; Ali et al. 2005), antibiotic (Abd El-Hack et al. 2020), antidiabetic (Vangalapati et al. 2012; serairi beji et al. 2018; Kumar et al. 2019), anti-inflammatory effects (Ho et al. 2013; Chao et al. 2005; Han and Parker 2017), anticancer (Kubatka et al. 2020) and other innumerable effects (Mehrpouri et al. 2020; Premakumara and Abeysekera 2020). Like cinnamon, cumin (Cu) (Cuminum *cyminum*) seeds are also used in traditional medicine for the treatment of various ailments. Cuminaldehyde, *α-* and *β-*pinene, limonene, *o-* and *p-*cymene, 1,8-cineole, *α-* and *γ-*terpinene, linanool and safranal are the main components of cumin (Rudra Pratap et al. 2017; Mnif and Aifa 2015; Singh et al. 2021; Koohsari et al. 2020; Srinivasan 2018) (Fig. 1).

Nowadays, poly(D, L-lactide-co-glycolide) (PLGA)-based nanotechnology has gained immense attention in medical applications as is easily broken down by hydrolysis into lactic acid and glycolic acid. The monomers are also biocompatible. It owes unique properties like bioavailability, biocompatibility, changing the kinetics of degradation, stability, increased drug loading and prolonged release of drug in comparison with other carriers. Also, these systems protect sensitive ingredients from unwanted reactions that lead to structural changes (Campos et al. 2016). These new drug delivery approaches, including polymeric nanoparticles (NPs), are projected to transform the view of pharmaceutical companies.

The present study emphasizes on the preparation of cinnamon and cumin (Cu–Ci) dual drug-loaded PLGA NPs with simple and cost-effective solution evaporation method. Till now, no attempts have been made to co-encapsulate these drugs and to study their combined potency. Based on the studies, the drug encapsulation efficiency was calculated to be 79% ± 4.5%. Moreover, the Ci-Cu-loaded polymeric NPs exhibited nearly two and three-times slower drug release rate compared to cinnamon-loaded and cumin-loaded polymeric NPs, respectively.

## Materials and methods

### Materials

PLGA [poly (D, L-lactide-co-glycolide)] with a 50:50 copolymeric ratio of DL-lactide to glycolide (Mw = 24,000 to 38,000 Da) was obtained from Sigma-Aldrich. Pluronic F-68 used as the surfactant was taken from Sigma-Aldrich. Analytical grade methanol and acetonitrile organic solvents were used. Disodium hydrogen phosphate (Na_2_HPO_4_), sodium dihydrogen phosphate (NaH_2_PO_4_), and trisodium phosphate (Na_3_PO_4_) used in this work were also analytical grade. For in vitro release studies, dialysis membrane (HiMedia, MWCO 12–14 KDa) with a pore size of 2.4 nm was used.

### Preparation of cinnamon and cumin extract

The bark of cinnamon and seeds of cumin was purchased from the market. The plant samples were further submitted to Raw Material Herbarium and Museum, Delhi (RHMD) for certification. Authentication for both the plants in the study was done as follows:

- Cuminum *cyminum*- NISCAIR/RHMD/Consult/2019/3422- 23–1.
- Cinnamomum *verum*- NISCAIR/RHMD/Consult/2019/3422- 23–2.

After that, the impurities were removed, and both spices were air-dried at room temperature and grounded in a mixer to a fine powder. The powder was sieved and stored in air-tight bottles to get an even size range. Extraction was done using methanol. Powdered samples (8 gm) were soaked for 72 h in 100 ml methanol in a sterile beaker covered with aluminum foil to avoid evaporation, and then it was filtered using filter paper (Whatman No. 1). The solvent from the filtrate was evaporated under room temperature conditions. Finally, the dried-up extract was kept in bottles till further usage.

### Formulation of PLGA nanoparticles loaded with cinnamon and cumin

**Scheme 1:**
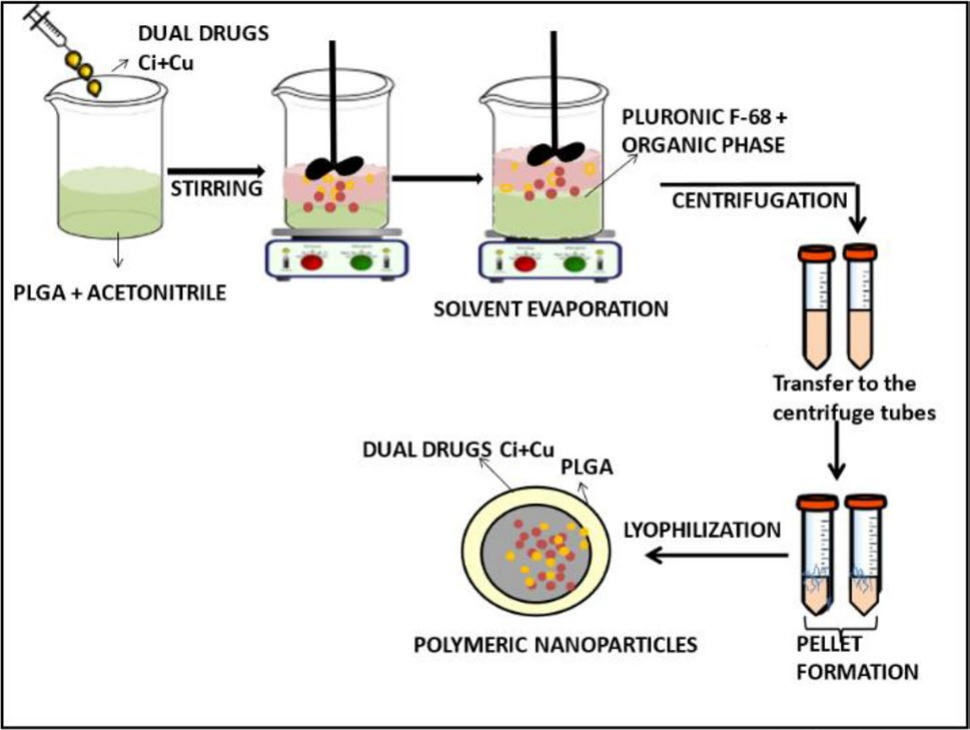
Schematic representing the synthesis of PLGA nanoparticles loaded with cinnamon and cumin by solvent evaporation method

Solvent evaporation method was adopted to formulate polymeric NPs with or without methanolic extract of cinnamon and cumin. Scheme 1 shows the schematic of PLGA nanoparticles loaded with cinnamon and cumin. To prepare the organic phase, 10 ml acetonitrile was added to 5 mg methanolic extracts of cinnamon and cumin, along with 50 mg of PLGA. The organic phase was added dropwise to an aqueous solution (25 ml of 0.1% Pluronic F-68). Further the mixture was sonicated in an ultrasonicator (Hielscher Ultrasonics, Germany) for 3 min. The organic solvent was evaporated from the resulting dispersion of nanoparticles by continuous stirring (400 rpm) at room temperature for 4 h followed by centrifugation (REMI, INDIA) at 15,000 rpm for 25 min. The obtained nanoparticles were washed several times with double distilled water followed by lyophilization. After that, the NPs were stored at 4 °C.

### Size determination

Nanoparticle size was determined using Dynamic Light Scattering (Zeatasizer Malvern Instruments, Worcestershire, UK). For measurements at scattering angle 90° and temperature 25 °C, 100 μl suspension of the nanoparticles was dispersed in 1 ml of distilled water and then sonicated for 1 min. The average diameter and the standard deviation were calculated for ten determinations of each sample.

### Evaluating the morphology of nanoparticles

#### SEM studies

The nanoparticle's surface morphology and shape were analyzed using scanning electron microscopy (SEM-Zeiss EVOMA10. Metal stubs were covered with double-sided adhesive tape, and a suitable nanoparticles sample was spread on the sticky surface. Vacuum dried samples were coated with a palladium layer through an ion sputter. Samples were observed after palladium coating of 30 nm at an accelerating voltage of 20 kV and 10 Pa.

#### TEM studies

The morphology of nanoparticles was further studied and compared employing transmission electron microscopy (TEM-JEOL-2100F, Japan). After dispersing the polymeric nanoparticles in distilled water, they were sonicated. The sample was prepared by placing a drop on a copper grid so that a thin liquid film was formed, which was allowed to dry before the examination, using a TEM without being stained.

#### XRD studies

PANalytical X-ray diffractometer at 40 mA anodic current was used to record the XRD patterns of cinnamon, cumin and cinnamon-cumin-loaded polymeric nanoparticles using an accelerating voltage of 45 kV. XRD patterns was obtained at a scanning speed 2° per minute were done at diffraction angles (2θ) from 5° to 90°.

#### FTIR studies

Fourier-transform infrared (FTIR) spectroscopy was used for sample analysis in the range 400–4000 cm^−1^ to validate the compatibility among polymeric, cinnamon-loaded polymeric nanoparticles, cumin-loaded polymeric nanoparticles and cinnamon-cumin-loaded polymeric nanoparticles. KBr was used to disperse the powdered samples and compress them into pellets.

### Determination of Ci–Cu entrapment efficiency

Drug encapsulation efficiency was calculated using UV–Visible spectroscopy. The supernatant collected after centrifugation was analyzed to check the amount of available drug in the nanoparticles. The absorbance of the drug was checked, and the presence was calculated using the standard curve of the drug prepared in water. The calibration curve for the quantification of cinnamon and cumin was linear over the range of standard concentrations from 0.1250 mg to 10 mg with a correlation coefficient of R^2^ = 0.998.

$${\text{Drug encapsulation efficiency}} \left( \% \right) = \frac{{{\text{Amount of drug added}} - {\text{Amount of drug in supernatant}} }}{{\text{Amount of drug added}}} \times 100$$

### In vitro release studies

At pH 6.9, phosphate-buffered saline (PBS) solution was used to carry out release studies of the drug up to 25 h from Ci-Cu-loaded polymeric NPs. About 10 mg each of cinnamon, cumin, cinnamon-loaded polymeric NPs, cumin-loaded polymeric NPs and Ci-Cu-loaded NPs were put in a dialysis membrane. Further dialysis membrane was then put into a beaker having 20 ml PBS. This setup was kept under magnetic stirring (100 rpm) at room temperature. At predetermined time gaps (30 min, 1 h, 2 h, 4 h, 8 h, 24 h) withdrawal of 3 ml solution was done. Shimadzu UV–Vis spectrophotometer was used to record absorbance for analyzing the drug release at a wavelength of 275.5 nm. The cumulative release from various formulations was also drawn as a function of time.

## Results

### Determination of nanoparticles size and morphology evaluation

**Fig. 2:**
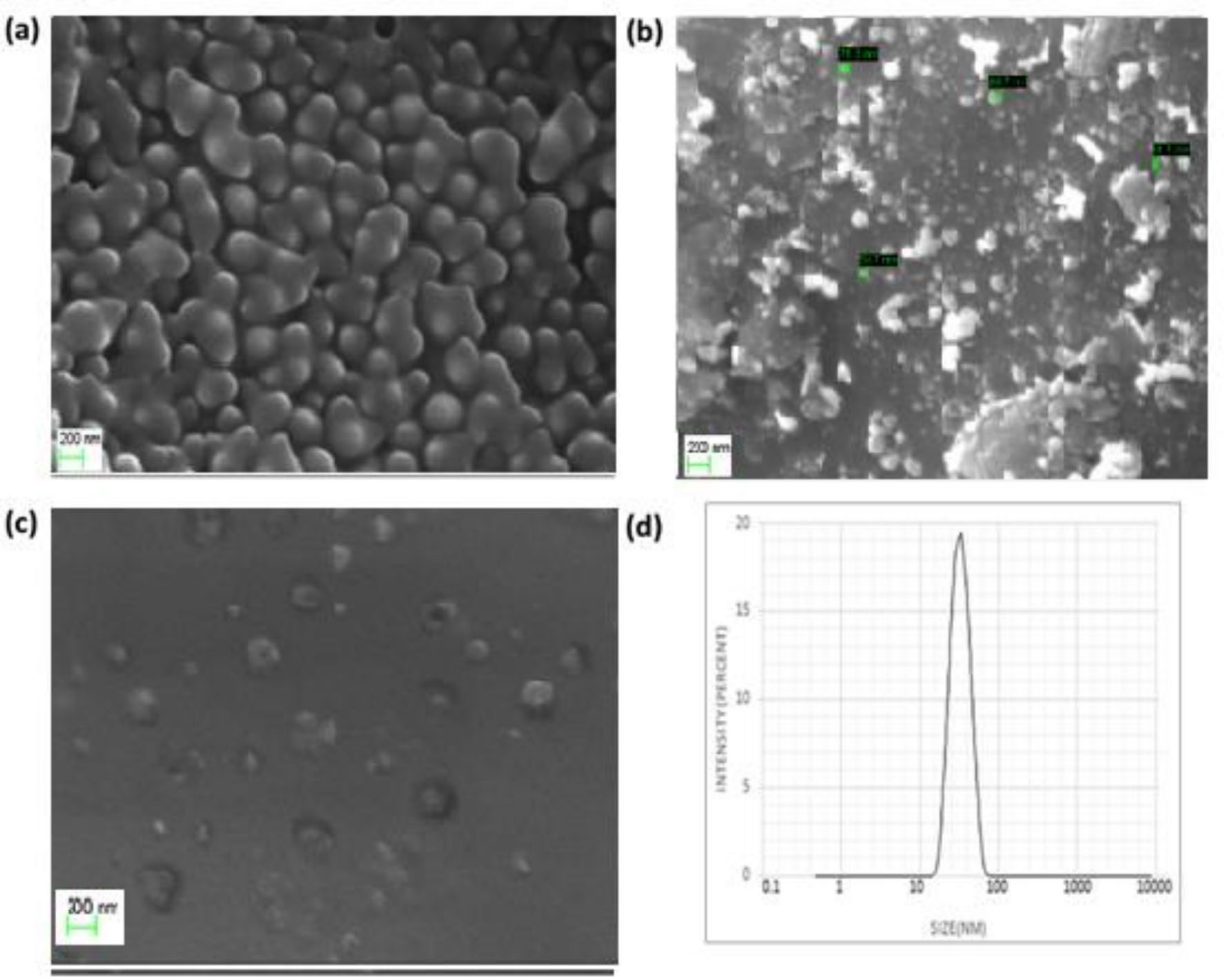
SEM images and size of nanoparticles loaded with **a** cinnamon **b** cumin **c** Ci-Cu. **d** Ci-Cu size distribution (scale bar is 200 nm)

**Fig. 3:**
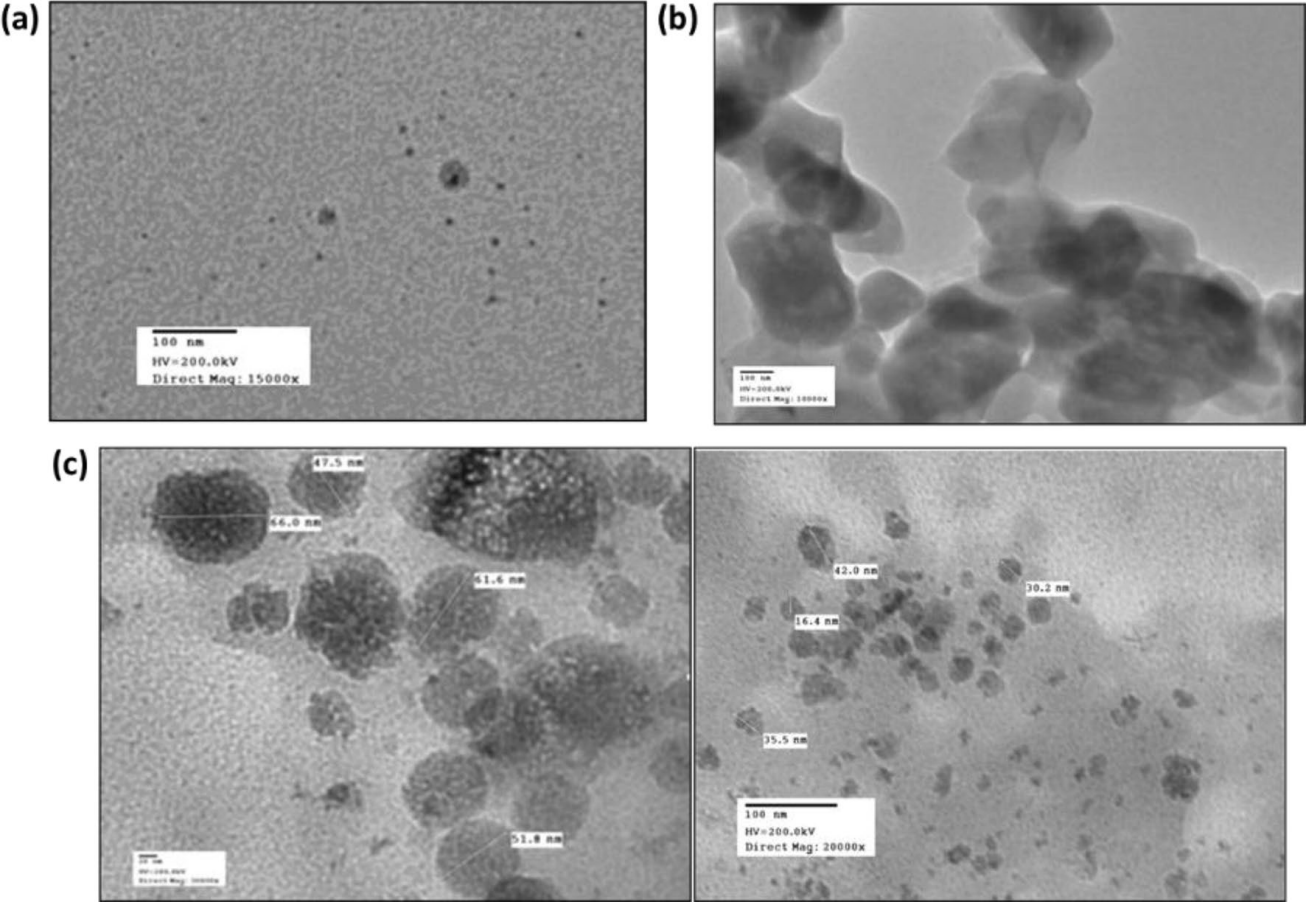
TEM images of nanoparticles loaded with **a** cinnamon **b** cumin **c** Ci–Cu (scale bar is 100 nm)

Figure 2(a–c) shows the morphology of cinnamon-loaded, cumin-loaded, and Ci-Cu-loaded nanoparticles. SEM studies indicated that distinctive spherical nanoparticles were formed. The TEM images of cinnamon-loaded, cumin-loaded and Ci-Cu-loaded polymer nanoparticles is shown in Fig. 3(a–c). TEM analysis also revealed the spherical shape of the synthesized nanoparticles. It was noticed that the size of the nanoparticles significantly decreased from 100 to 270 nm to 100–180 nm in cinnamon- and cumin-loaded PLGA nanoparticles. Whereas a size distribution of 90–120 nm was observed in the Cu–Ci dual drug-loaded polymeric NPs. It is expected that Ci-Cu-loaded NPs' decreased size can contributes majorly to slow drug release.

Entrapment efficiency is an important parameter, as inappropriate entrapment would lead to an initial burst release of the drug, which deters its sustained-release property. Based on the studies, the drug encapsulation efficiency was calculated to be 79% ± 4.5%.

### XRD Studies

**Fig. 4:**
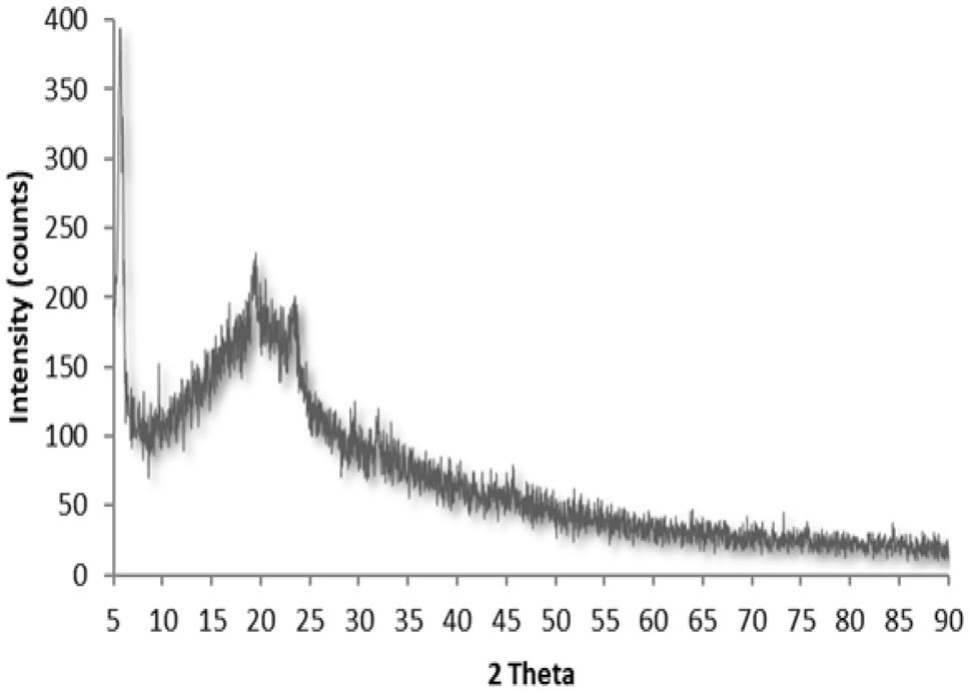
XRD diffractograms of Ci-Cu-loaded polymeric nanoparticles

The XRD patterns for Ci-Cu-loaded PLGA nanoparticles is shown in Fig. 4. For the drugs entrapped in polymeric nanoparticles, no characteristic peak was shown, possibly owing to the amorphous nature of the PLGA.

### FTIR studies

**Fig. 5:**
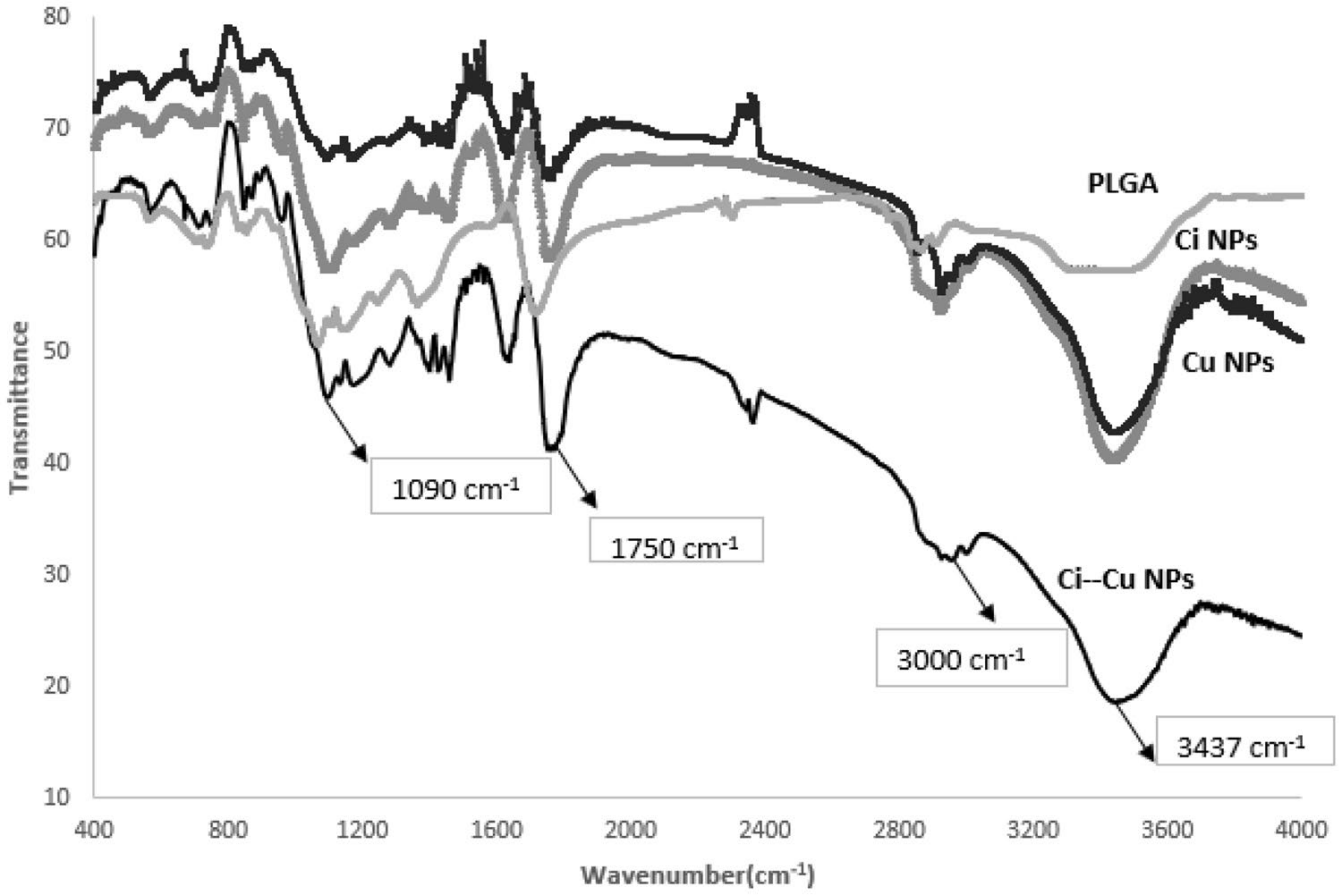
FTIR spectra of PLGA, cinnamon-loaded, cumin-loaded and Ci-Cu-loaded polymeric NPs

FTIR spectroscopic studies were done to analyze drugs' structural characteristics, PLGA and Ci-Cu-loaded nanoparticles, as shown in Fig. 5. At 3394 cm^−1^, 2942 cm^−1^, 1708 cm^−1^, 1442 cm^−1^, 1344 cm^−1^, 1346 cm^−1^, 1149 cm^−1^, 1051 cm^−1^, 730 cm^−1^ and 548 cm^−1^ bands of PLGA were observed. Besides the distinctive groups of PLGA, the Ci-Cu-loaded polymeric nanoparticles spectra also showed the bands for characteristic functional groups of cinnamon and cumin at 3437 cm^−1^, 3000 cm^−1^, 1750 cm^−1^, 1257 cm^−1^, 1143 cm^−1^, 1090 cm^−1^, 873 cm^−1^ and 665 cm^−1^. No peak shift or functional peaks loss of cinnamon and cumin peaks was noticed in Ci-Cu-loaded nanoparticles, indicating no chemical interactions between cinnamon and cumin drugs and the polymer.

### In vitro release studies

**Fig. 6:**
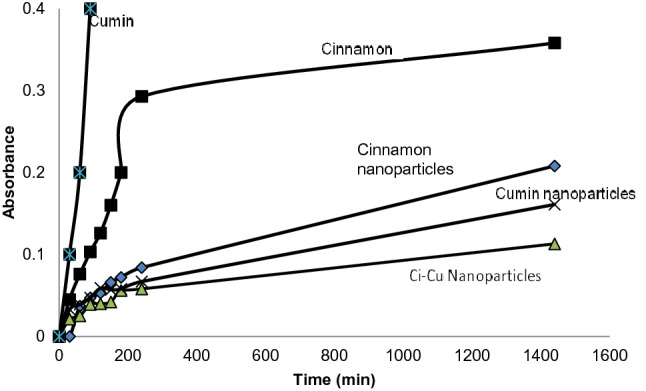
Absorbance spectra demonstrating comparative sustained release of cinnamon and cumin from polymeric nanoparticles

**Fig. 7:**
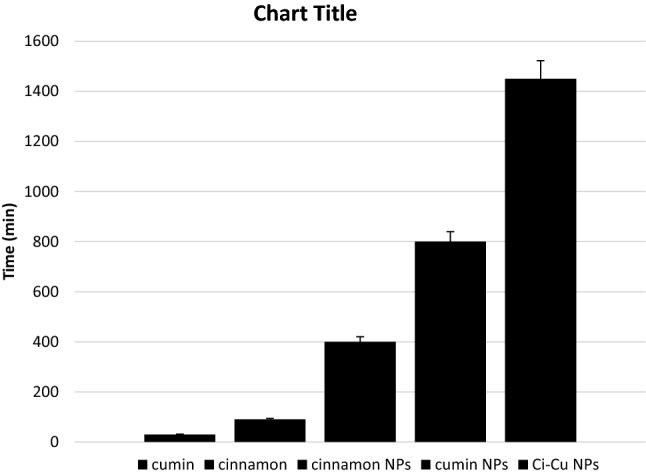
Comparative results of 25% release of the drug from various formulations as a function of time

The release profiles under in vitro conditions were studied of cinnamon, cumin, cinnamon-loaded polymeric NPs, cumin-loaded polymeric NPs and Ci-Cu-loaded polymeric Nps by UV–Vis spectroscopy (see Fig. 6). A biphasic release profile was shown by the drug-loaded PLGA nanoparticulate formulation. Various studies have reported the exponential release pattern for PLGA nanoparticles (Saxena et al. 2004; Brandt et al. 2021). The release profile of all formulations of PLGA nanoparticles exhibited a faster exponential release in the initial phase followed by a slower release phase. The results indicated that the Cu–Ci dual drug-loaded polymeric NPs has drug release at a slower rate. Figure 7 indicates that the time taken for 25% drug release from Ci-Cu-loaded polymeric NPs increases significantly as compared to cinnamon-loaded polymeric nanoparticles and cumin-loaded polymeric nanoparticles. The time taken for 25% release of drug in Ci-Cu-loaded PLGA nanoparticles is twice as compared to cumin-loaded PLGA nanoparticles, and three times compared to cinnamon-loaded PLGA nanoparticles.

## Conclusion

Cinnamon and cumin (Ci–Cu) dual drug-loaded PLGA polymeric nanoparticles were successfully synthesized by solvent evaporation method. The spherical nanoparticles prepared have size distribution of 90–120 nm with a good encapsulation efficiency of 79% ± 4.5%. The in vitro drug release studies showed the controlled biphasic release of drugs from Ci-Cu-loaded polymeric nanoparticles. The Ci-Cu-loaded polymeric nanoparticles exhibited nearly two- and three-times slower drug release rate compared to cinnamon-loaded and cumin-loaded polymeric NPs, respectively. The reported results provided a simple method for synthesizing polymeric nanoparticles loaded with cinnamon and cumin to cure numerous ailments.

## References

[CR1] Abd El-Hack ME, Alagawany M, Abdel-Moneim A-ME, Mohammed NG, Khafaga AF, Bin-Jumah M (2020). Cinnamon (Cinnamomum *zeylanicum*) Oil as a Potential Alternative to Antibiotics in Poultry. Antibiotics.

[CR2] Abdallah E, Rauf A, Sadeek AMM (2020). Cinnamon bark as antibacterial agent: A mini-review. GSC Biolog Pharmaceut Sci.

[CR3] Ali SM, Khan AA, Ahmed I, Musaddiq M, Ahmed KS, Polasa H (2005). Antimicrobial activities of Eugenol and Cinnamaldehyde against the human gastric pathogen Helicobacter pylori. Ann Clin Microbiol Antimicrob.

[CR4] Anand V, Kumar S, Manickam AH (2016). Cinnamomum *zeylanicum* Linn. The spice with multi potential. Systemat Rev Pharm.

[CR5] Błaszczyk N, Rosiak A, Kałużna-Czaplińska J (2021). The potential role of cinnamon in human health. Forests.

[CR6] Brandt JV, Piazza RD, Carvalho dos Santos C, Vega-Chacón J, Amantéa BE, Pinto GC, Jafelicci M, Marques RFC (2021). Synthesis of core@shell nanoparticles functionalized with folic acid-modified PCL-co-PEGMA copolymer for methotrexate delivery. Nano-Str Nano-Objects.

[CR7] Chao LK, Hua K-F, Hsu H-Y, Cheng S-S, Liu J-Y, Chang S-T (2005). Study on the antiinflammatory activity of essential oil from leaves of cinnamomum *osmophloeum*. J Agric Food Chem.

[CR8] Doyle AA, Stephens JC (2019). A review of cinnamaldehyde and its derivatives as antibacterial agents. Fitoterapia.

[CR9] Gulcin I, Kaya R, Goren AC, Akincioglu H, Topal M, Bingol Z (2019). Anticholinergic, antidiabetic and antioxidant activities of cinnamon (cinnamomum *verum*) bark extracts: polyphenol contents analysis by LC-MS/MS. Int J Food Prop.

[CR10] Hamidpour R, Hamidpour M, Hamidpour S, Shahlari M (2015). Cinnamon from the selection of traditional applications to its novel effects on the inhibition of angiogenesis in cancer cells and prevention of Alzheimer's disease, and a series of functions such as antioxidant, anticholesterol, antidiabetes, antibacterial, antifungal, nematicidal, acaracidal, and repellent activities. J Tradit Complem Med.

[CR11] Han X, Parker TL (2017). Antiinflammatory activity of cinnamon (Cinnamomum *zeylanicum*) bark essential oil in a human skin disease model. Phytotherapy Res.

[CR12] Ho S-C, Chang K-S, Chang P-W (2013). Inhibition of neuroinflammation by cinnamon and its main components. Food Chem.

[CR13] Kallel I, Hadrich B, Gargouri B, Chaabane A, Lassoued S, Gdoura R (2019). Optimization of Cinnamon (Cinnamomum *zeylanicum* Blume) Essential Oil Extraction: Evaluation of Antioxidant and Antiproliferative Effects. Evidence Based Complement Alternat Med.

[CR14] Koohsari S, Sheikholeslami MA, Parvardeh S, Ghafghazi S, Samadi S, Poul YK (2020). Antinociceptive and antineuropathic effects of cuminaldehyde, the major constituent of Cuminum *cyminum* seeds: Possible mechanisms of action. J Ethnopharmacol.

[CR15] Kubatka P, Kello M, Kajo K, Samec M, Jasek K, Vybohova D (2020). Chemopreventive and Therapeutic Efficacy of Cinnamomum *zeylanicum* L. bark in experimental breast carcinoma: mechanistic in vivo and in vitro analyses. Molecules.

[CR16] Kumar S, Kumari R, Mishra S (2019). Pharmacological properties and their medicinal uses of Cinnamomum: a review. J Pharm Pharmacol.

[CR17] Mnif S, Aifa S (2015). Cumin (Cuminum cyminum L.) from Traditional Uses to Potential Biomedical Applications. Chem Biodiver.

[CR18] Paswan VKS, Kukreja G, Bunkar DS, Bhinchhar BK (2021). Health Benefits and Functional and Medicinal Properties of Some Common Indian Spices.

[CR19] Saxena V, Sadoqi M, Shao J (2004). Indocyanine green-loaded biodegradable nanoparticles: preparation, physicochemical characterization and *in vitro* release. Int J Pharm.

[CR20] SerairiBeji R, Sameh K, AidiWannes W, Ayari K, Riadh K (2018). Antidiabetic, antihyperlipidemic and antioxidant influences of the spice cinnamon (Cinnamomum *zeylanicumon*) in experimental rats. Brazilian J Pharmaceut Sci.

[CR21] Singh N, Yadav SS, Kumar S, Narashiman B (2021). A review on traditional uses, phytochemistry, pharmacology, and clinical research of dietary spice Cuminum *cyminum* L. Phytotherapy Res.

[CR22] Srinivasan K (2018). Cumin (Cuminum *cyminum*) and black cumin (Nigella *sativa*) seeds: traditional uses, chemical constituents, and nutraceutical effects. Food Quality Safety.

[CR23] Vangalapati M, Satya N, Prakash DV, Avanigadda S (2012). A review on pharmacological activities and clinical effects of Cinnamon species. Res J Pharm Biol Chem Sci.

[CR24] Yashin A, Yashin Y, Xia X, Nemzer B (2017). Antioxidant activity of spices and their impact on human health: a review. Antioxidants.

[CR25] Ajay A, Basalingappa K, Nataraj R, Chettiyar B 13 (2020) Phytochemical screening and antimicrobial activity of "Cinnamon *zeylanicum*"'. Int J Pharm Res Innov pp. 22–33

[CR26] Campos PM, Bentley MVLB, Torchilin VP (2016) Chapter 1 - Nanopreparations for skin cancer therapy. In: Grumezescu AM (Ed.), Nanobiomaterials in Cancer Therapy (pp. 1–28). William Andrew Publishing. 10.1016/B978-0-323-42863-7.00001-3.

[CR27] Mehrpouri M, Hamidpour R, Hamidpour M (2020) Cinnamon inhibits platelet function and improves cardiovascular system. JMPIR 19(73): 1–11. 10.29252/jmp.1.73.1.

[CR28] Premakumara GAS, Abeysekera WPKM (2020) Pharmacological properties of Ceylon Cinnamon. In: Senaratne R, Pathirana R (Eds.), Cinnamon: Botany, Agronomy, Chemistry and Industrial Applications (pp. 307–325). Springer International Publishing, Cham. 10.1007/978-3-030-54426-3_12.

[CR29] Rudra Pratap S, Gangadharappa HV, Mruthunjaya K (2017) Cuminum *cyminum* – A popular spice: An updated review. Pharmacognosy J /files/PJ-9-3/10.5530pj.2017.3.51

